# Low Temperature Aluminothermic Reduction of Natural Sepiolite to High-Performance Si Nanofibers for Li-Ion Batteries

**DOI:** 10.3389/fchem.2022.932650

**Published:** 2022-06-27

**Authors:** Mingyuan Zhao, Shaobin Yang, Wei Dong

**Affiliations:** ^1^ College of Mines, Liaoning Technical University, Fuxin, China; ^2^ College of Materials Science & Engineering, Liaoning Technical University, Fuxin, China

**Keywords:** sepiolite, silicon, nanofiber, aluminothermic reduction, lithium-ion battery, anode materials

## Abstract

Nanostructure silicon is one of the most promising anode materials for the next-generation lithium-ion battery, but the complicated synthesis process and high cost limit its large-scale commercial application. Herein, a simple and low-cost method was proposed to prepare silicon nanofibers (SNF) using natural sepiolite as a template *via* a low-temperature aluminum reduction process. The low temperature of 260°C during the reduction process not only reduced the production cost but also avoided the destruction of the natural sepiolite structure caused by the high temperature above 600°C in the traditional magnesium thermal reduction process, leading to a more complete nanofiber structure in the final product. For the first time, the important role of Mg-O octahedral structure in the maintenance of nanofiber structure during the process of low-temperature aluminothermic reduction was verified by experiments. When used as an anode for lithium-ion batteries, SNF yield a high reversible capacity of 2005.4 mAh g^−1^ at 0.5 A g^−1^ after 50 cycles and 1017.6 mAh g^−1^ at 2 A g^−1^ after 200 cycles, remarkably outperforming commercial Si material. With a low-cost precursor and facile approach, this work provides a new strategy for the synthesis of a commercial high-capacity Si anode.

## Introduction

Lithium-ion batteries with the advantages of high energy density, environmental friendliness, and long cycle life have been widely used in portable electronic devices and occupy most of the energy storage market of hybrid electric vehicles (Dunn et al., 2011; Lin et al., 2015; Luo et al., 2015; Hong et al., 2020a, 2020b). The theoretical capacity of graphite anode materials commonly used in lithium-ion batteries was low, which could not meet the growing demand for large-scale energy storage and hybrid electric vehicles. Owing to the characteristics of high theoretical capacity (4300 mAh g^−1^) and low working potential (<0.4V vs. Li/Li^+^) (Lin et al., 2016), silicon (Si) was regarded as a promising anode material for next-generation lithium-ion batteries. However, the huge volume change of the Si anode during the process of Li^+^ intercalation and deintercalation led to the continuous cracking and crushing of the active material, which eventually led to the continuous decline of the capacity of the Si anode (Pan et al., 2018). Meanwhile, Si material had low intrinsic conductivity and poor rate performance when used as an anode material. The above shortcomings hindered the commercial application of Si anode materials (Zuo et al., 2017).

In order to solve these problems, a large number of nanostructures, such as nanowires (Dong et al., 2017; Hwang et al., 2017), nanotubes (Wan et al., 2018), and hollow nanospheres (Chen et al., 2019), have been developed to suppress volume expansion and improve structural stability during cycling. One-dimensional (1D) nano Si had two advantages due to its special structure. First, 1D nanostructure with strong charge transport along the axis could promote lithium-ion diffusion and effectively improve the rate performance of materials (Jiang et al., 2017). Second, 1D nanostructure allowed radial volume expansion to minimize the tendency of cracking (Zuo et al., 2017), thus improving its cyclic properties. The results showed that 1D nanostructured Si anode exhibited excellent performance in lithium-ion batteries.

The traditional preparation methods of one-dimensional nanostructured Si, including chemical vapor deposition (CVD) (Huang et al., 2014; Epur et al., 2015; Hou et al., 2017) and the electrochemical method (Yang et al., 2009; Bard et al., 2019; Yang et al., 2019) of Si precursors, were complex and high-cost, which were not suitable for commercial production of high-quality and controllable morphology of one-dimensional nanostructures. Recently, direct synthesis of one-dimensional nano Si *via* reduction of natural mineral precursors has been regarded as an environmentally friendly, low-cost, and simple route. Popular reduction methods include carbothermal reduction and magnesiothermic reduction, in which magnesium thermal reduction was a common reduction method due to its relatively low reduction temperature (500–900°C) and controllable morphology (Furquan et al., 2018; Tang et al., 2018; Feng et al., 2019; Zhang et al., 2019). However, Mg_2_Si and Mg_2_SiO_4_ were inevitably produced in the process of magnesiothermic reduction, resulting in the introduction of impurities into the products (Sandhage et al., 2002), which had a great impact on the electrochemical performance. In addition, the high temperature above 500°C could easily lead to the sintering of the products, which would destroy the structure of the Si source itself and lead to the serious agglomeration of the products (Wang et al., 2017; Zhuang et al., 2017).

In order to obtain high-quality one-dimensional nano Si and reduce the energy consumption during the experiment, a molten salt system for low-temperature reduction of silica (SiO_2_) was used in this study (Gao et al., 2018). Finally, one-dimensional Si nanofibers (SNF) were successfully prepared by aluminothermic reduction of natural sepiolite in molten AlCl_3_. The related chemical reaction could be expressed as 4Al + 3SiO_2_ + 2AlCl_3_→ 3Si + 6AlOCl (Lin et al., 2015; Chen et al., 2018; Chen et al., 2019). The inherent fibrous structure of sepiolite was maintained during the low-temperature aluminothermic reduction process. The AlOCl produced by the reaction could be easily removed with HCl to obtain high-purity SNF. In addition, sepiolite without Mg-O octahedral structure (obtained by washing with excess hydrochloric acid) was subjected to the same low-temperature aluminothermic reduction process. To the best of our knowledge, the significant role of Mg-O octahedron in maintaining the fibrous structure of sepiolite during the low-temperature aluminothermic reduction process was verified by experiments for the first time. When used as anode materials for lithium-ion batteries, SNF exhibits excellent electrochemical properties, including excellent rate capability (2921.6 mAh g^−1^ at 0.1 A g^−1^ and 1633.6 mAh g^−1^ at 2 A g^−1^) and significant cycle stability (capacity retention of 1197.6 mAh g^−1^ at 2.0 A g^−1^ after 200 cycles). The method had broad prospects in commercial production owing to its abundant sources of raw materials, low cost, and simple preparation process.

## Materials and Methods

### Materials Synthesis

First, the sepiolite powder (Xiangtan Sepiolite Technology Co., Ltd., 300 mesh) was cleaned with deionized water and dried at 80°C for 10 h. The chemical composition of sepiolite is given in Table 1. The pretreated natural sepiolite powder (1 g), aluminum powder (0.4 g) (Sinopharm, 100 mesh), and AlCl_3_ powder (4 g) (Sinopharm, analytical pure) were finely ground and mixed evenly and then sealed in a stainless reactor in an argon atmosphere glove box. Then the reactor was placed in a blast drying box, heated to 200°C, 260°C or 300°C at a temperature increase rate of 5°C/min, and maintained at a constant temperature for 10 h. After cooling down to room temperature at a cooling rate of 2°C/min, the mixture was washed with 1M HCl solution (Sinopharm, wt. 33.6%) for 5 h to remove reaction by-products (AlOCl) and other impurities (excess Al powder and other mineral impurities of sepiolite). Then, it was added into 5 wt.% HF (Sinopharm, 10 vol.%)/EtOH (Sinopharm) solution and stirred for 15 min. Finally, the SNF products were collected after being washed with deionized water. Natural sepiolite powder (1g) was treated with 1M HCl solution for 5 h to remove Mg-O octahedral structure which existed in the form of Mg_8_Si_12_O_30_(OH)_4_(H_2_O)_4_·8H_2_O at 25°C. Then, pre-pickling sepiolite was obtained after being washed with deionized water and dried. The reduction of commercial SiO_2_ (Sinopharm, analytical pure, 300 mesh) and pre-pickling sepiolite was carried out at 260°C with the same weight ratio of the reagents including metallic Al and AlCl_3_.

**TABLE 1:** Chemical composition of sepiolite.

Chemical composition	Mass fraction (%)
SiO_2_	52.41 (±0.12)
MgO	16.19 (±0.09)
CaO	8.33 (±0.06)
H_2_O	6.43 (±0.01)
Fe_2_O_3_	1.47 (±0.01)

### Characterization

The microscopic morphology and element distribution of the sample were observed using the SU-8010 cold field emission scanning electron microscope (Hitachi, Japan). The powder of the sample to be tested after ultrasonic dispersion adhered to the conductive glue on the sample stage, and the scanning electron microscope (SEM) images were tested after spray metal plating of Pt. Transmission electron microscopy (TEM) was used to analyze the microstructure, high-resolution lattice images and selected area electron diffraction patterns of the samples. The test instrument model was Joel JEM-100CX. The sample powder was first dispersed evenly with water or ethanol, and then dropped on the surface of the carbon support film or microgrid, and dried for testing. X-ray diffraction (XRD) was tested on Bruker D8 Advance diffractometer (Bruker AXS, Germany), operating at 40 kV of tube voltage and 40 mA of current with Cucd as the anode target. The diffraction angle scan range was 5–80° theta, and the scan speed was 6° min^−1^. The Raman spectrum analysis instrument adopted Bruker Optics Senterra R200-L with Ar ion laser as a light source, the excitation wavelength was 532 nm, and the data acquisition time was 10 s. The XPS spectrometer was an AXIS UltraDLD electronic spectrometer from Shimadzu Kratos, Japan. The test conditions were the AlKα target (1486.6 eV), with the vacuum degree lower than 5 × 10^–9^ Torr. The binding energy was corrected with C 1s = 284.8 eV, and the peak fitting was performed by Casa XPS software. N_2_ adsorption-desorption isotherms of sepiolite and SNF were collected through the ASAP 2020 system (Micromeritics, USA) at liquid nitrogen temperature (−196°C). Before measurement, the sample was deflated at 200°C vacuum for 12 h. The specific surface area was calculated by the multipoint Brunauer Emmett Teller (BET) equation, and the total pore volume was obtained according to the N_2_ adsorption capacity at the relative pressure of 0.97. The pore size distribution was calculated according to the Barrett–Joyner–Halenda (BJH) method.

### Electrochemical Measurements

The electrochemical properties of SNF were tested in a coin-type half-cell (CR 2025). The working electrode preparation method was as follows: The active material, conductive agent acetylene black (super P), and binder sodium alginate (SA) were uniformly mixed in deionized water at a mass ratio of 6:2:2. After magnetic stirring for 4 h, the slurry was prepared by high-speed shearing for 30 min. The slurry was evenly coated on the ethanol, cleaned and dried Cu foil, and then vacuum dried at 60°C for 5 h. For coin-cell fabrication, a lithium metal plate was used as a counter electrode and polyethylene membranes (Entek et20-26) as a separator. 1 M LiPF_6_ solution (EC/DMC, 1:1 v/v) containing 10 vol.% FEC addition was used as an electrolyte. The battery was assembled in a glove box filled with Ar gas. The cycle performance and rate performance of the battery were tested by NEWARE 3008 test system. The cut-off voltage of the charge–discharge test was 0.001–2 V, the electrode activation current density was 200 mA g^−1^, and the cycle current density was 500 mA g^−1^, and 2 A g^−1^. Rate performance was measured in the current density range of 0.1–2 A g^−1^. The specific capacity of the battery was calculated according to the mass of the active material. Cyclic voltammetry (CV) and electrochemical impedance spectroscopy (EIS) were performed on CHI 660E electrochemical workstation. The scanning speed of cyclic voltammetry was 0.1 mV s^−1^ and the cut-off voltage was 0–1.5 V. The frequency range of the electrochemical impedance spectroscopy test was 0.01–100 kHz, and the amplitude was 5 mV. All electrochemical tests were performed at 25°C.

## Results and Discussion

**FIGURE 1:**
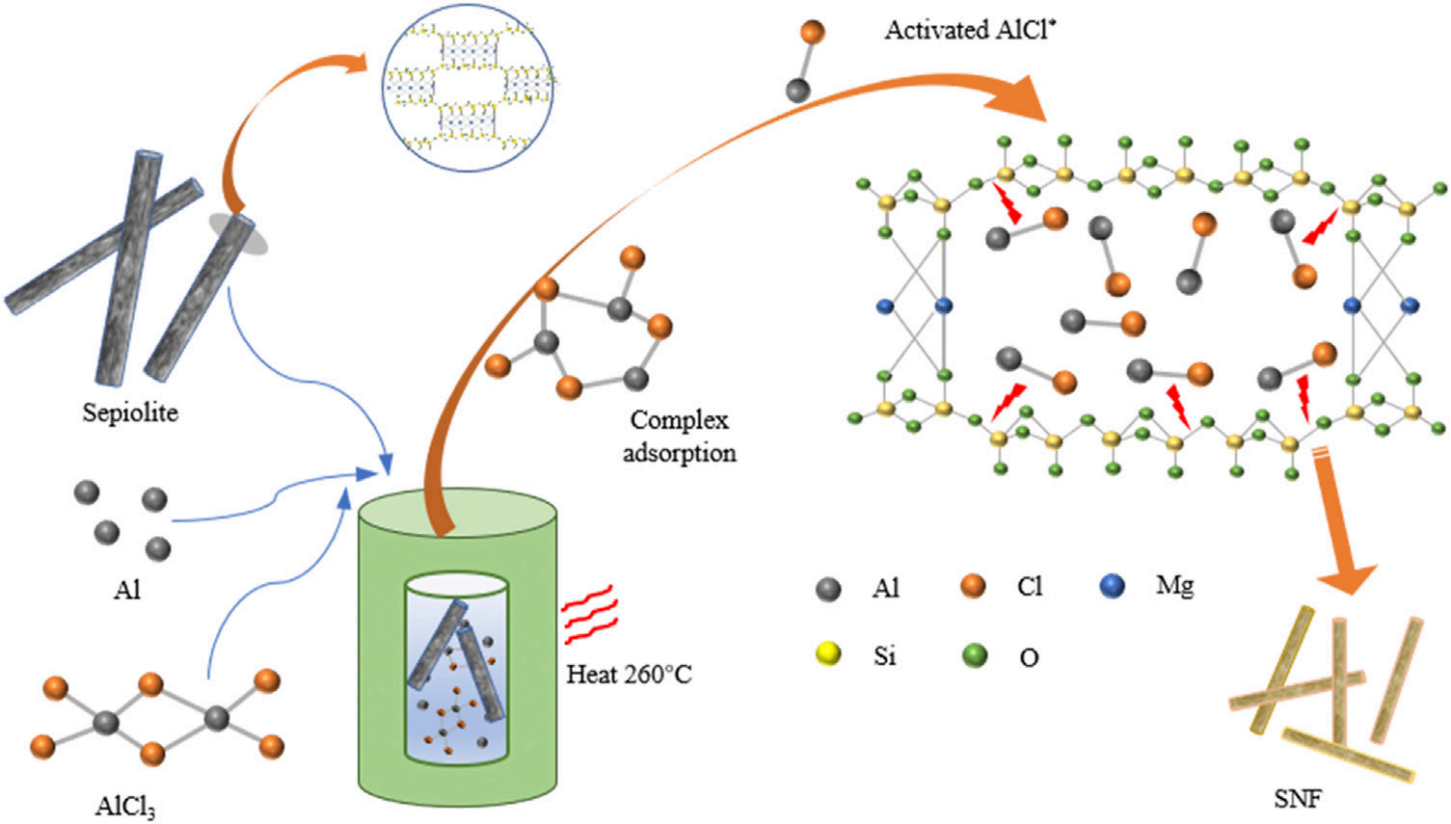
Schematic of SNF from natural sepiolite.

The process of preparing SNF from natural sepiolite by low-temperature aluminothermic reduction was exhibited (Figure 1). Sepiolite is a kind of clay mineral with a chain structure of a 2:1 layer, which consists of two layers of continuous silica tetrahedron and a discontinuous layer of Mg-O octahedron. The Si-O-Si bond and three-dimensional bond structure in sepiolite can combine the molecular chain, and the crystal morphology grows along a certain direction, and finally shows a fibrous morphology. Meanwhile, nanochannels (0.37 × 1.06 nm^2^) parallel to the fiber extension direction form between the silica tetrahedral layers owing to the discontinuous octahedral layer of magnesium atoms in sepiolite (Brigatti et al., 2006). The low-temperature aluminothermic reduction reaction was initiated above 194°C. When the temperature exceeded the melting point of AlCl_3_, Al-AlCl_3_ complexes were formed *in situ* on the surface of Al metal atoms through solvation. Then, the complex entered into the sepiolite nanochannel and adsorbs on the surface of the silica tetrahedral layer to form activated AlCl*. The activated AlCl* formed *in situ* on the surface of SiO_2_ can break the Si-O bond and detach the O atoms, and finally form AlOCl and Si (Song et al., 2018).

**FIGURE 2:**
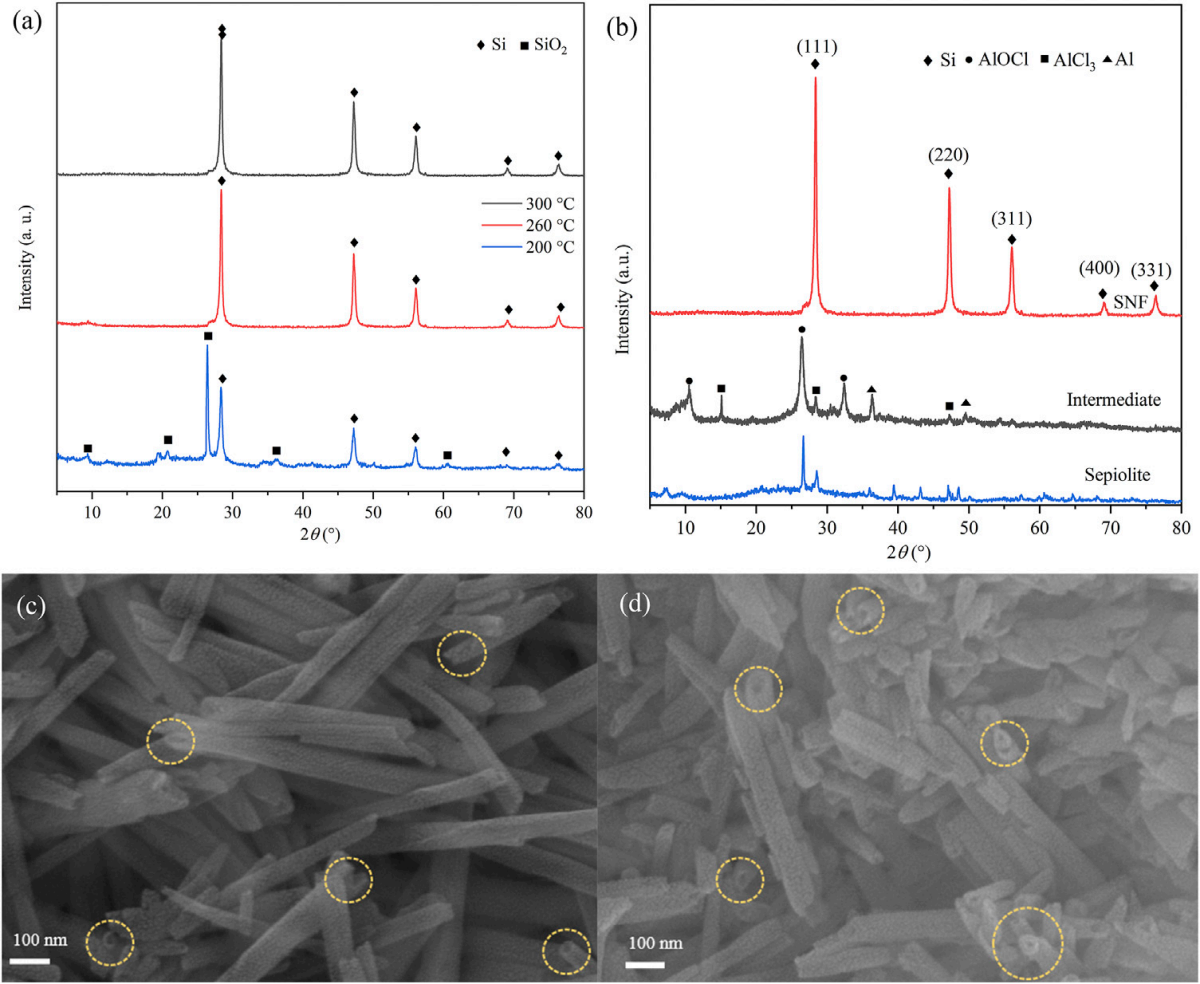
XRD patterns of products in different **(A)** reaction temperatures and **(B)** reaction stages. SEM images of **(C)** natural sepiolite and **(D)** SNF showing 1D nanofiber morphology (Yellow ring marks material pores).

The effect of reaction temperature on low-temperature aluminothermic reduction was studied by analyzing the XRD results of reduction products (Figure 2A) at different temperatures. The products were washed with 0.1 M hydrochloric acid (HCl), distilled water, and ethanol, but HF solution was not used. When the reduction temperature was 200°C, the diffraction peaks of SiO_2_ and Si existed simultaneously, which indicated that sepiolite could only be partially reduced. When the reduction temperature was raised to 260°C, the SiO_2_ peak disappeared completely, which meant that SiO_2_ in sepiolite was reduced more thoroughly. The yield of SNF which was calculated after SiO_2_ was removed by HF increased from 35% at 200°C to 75% at 260°C. However, the yield did not increase significantly after the temperature was raised to 300°C. The products at 260°C were taken as samples for the next analysis.

The XRD images of products in each reaction stage are shown in Figure 2B. Natural sepiolite, which served as a template, presented multiple sharp diffraction peaks and showed obvious crystal structure. The XRD results of the intermediate after low-temperature aluminothermic reduction showed that the SiO_2_ diffraction peak of sepiolite disappeared, and the AlOCl diffraction peak could be observed, which proved that the SiO_2_ in sepiolite was completely reduced and produced by-products during the low-temperature aluminothermic process. In addition, the diffraction peaks of Al and AlCl_3_ appeared in the intermediate products, which indicated that the amount of Al and AlCl_3_ added in the initial stage of the experiment was excessive in order to ensure the complete reduction of SiO_2_. For the final products, SNF after HCl and HF pickling, only Si diffraction peaks corresponding to (111), (220), (311), (400), and (331) crystal planes were observed at 28.26°, 47.35°, 56.44°, 69.57°, and 76.21°, respectively, which indicated that the pickling process effectively removes AlOCl which produced during aluminothermic reduction reaction and other impurities.

**FIGURE 3:**
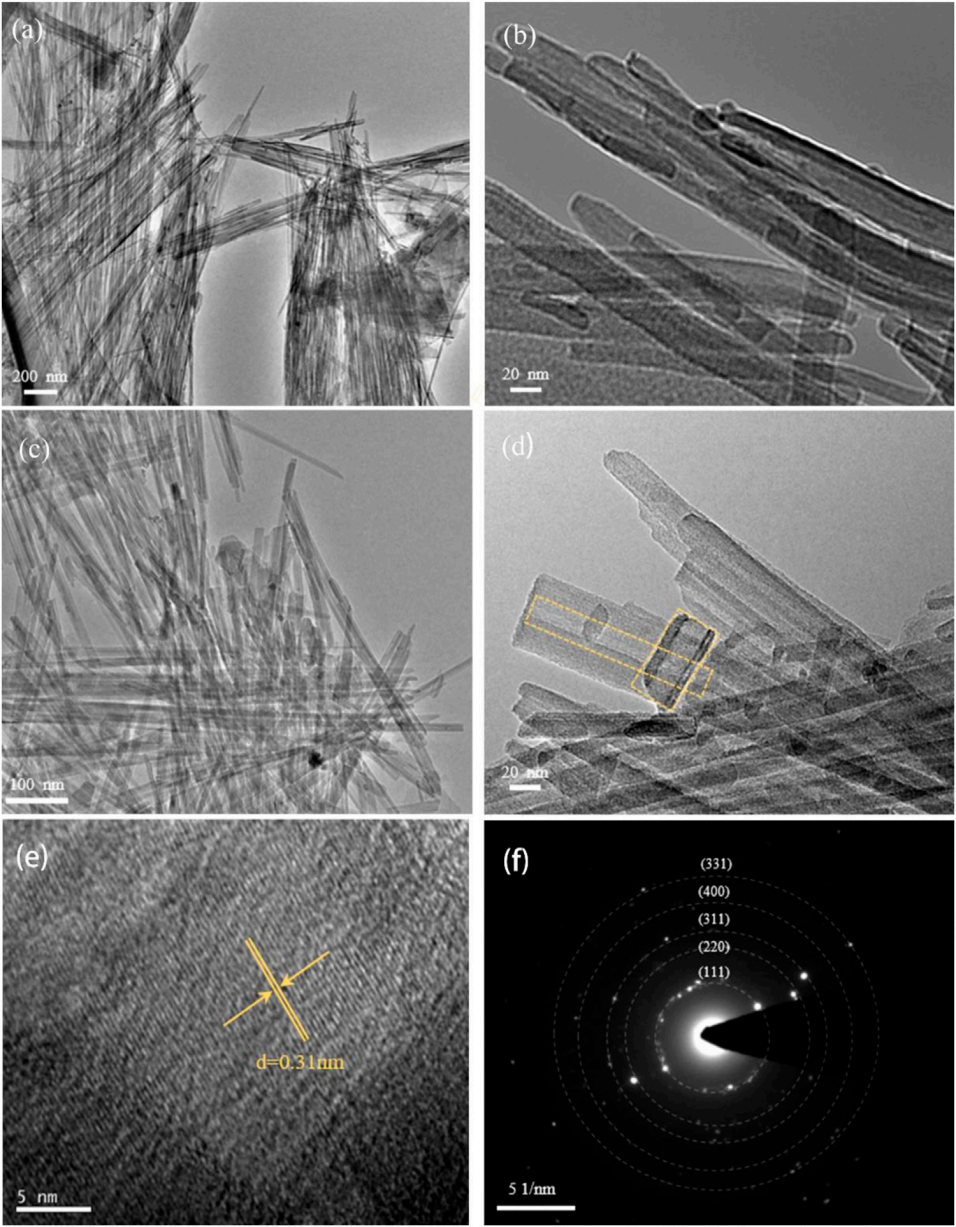
**(A)** TEM and **(B)** magnified TEM of natural sepiolite showing 1D nanofiber morphology. Morphological characterization of SNF, **(C)** TEM, **(D)** magnified TEM, **(E)** HRTEM, and **(F)** the corresponding SAED pattern (Yellow ring marks material pores).

The SEM images of sepiolite and SNF (Figures 2C and D) showed that sepiolite was composed of micro- and nano-sized fiber bundles. Compared with natural sepiolite materials, the final product still retained the original one-dimensional fibrous structure of sepiolite, but the fiber bundle presented a rough surface, and some fibers tended to become shorter and finer. The reason for this phenomenon was that the addition of HCl removed the impurities on the surface of sepiolite, which was consistent with the results of XRD. Compared with natural sepiolite (Figures 3A and B), TEM results in Figures 3C and D further confirmed that SNF had a one-dimensional nanofiber structure where the fiber structure was obvious, and the internal pore structure could be observed more clearly, which indicated that sepiolite acted as a template and precursor at the same time during the formation of one-dimensional nanofibers. Moreover, a hierarchical porous structure was observed in TEM images. In addition to the nanopores existing in the sepiolite fiber, macropores were formed during the disordered accumulation of the fibers. HRTEM (Figure 3E) and SAED (Figure 3F) images of SNF showed that the Si fibers were composed of crystalline Si, and the lattice spacing of 0.31 nm corresponded to the (111) crystal plane of Si.

**FIGURE 4:**
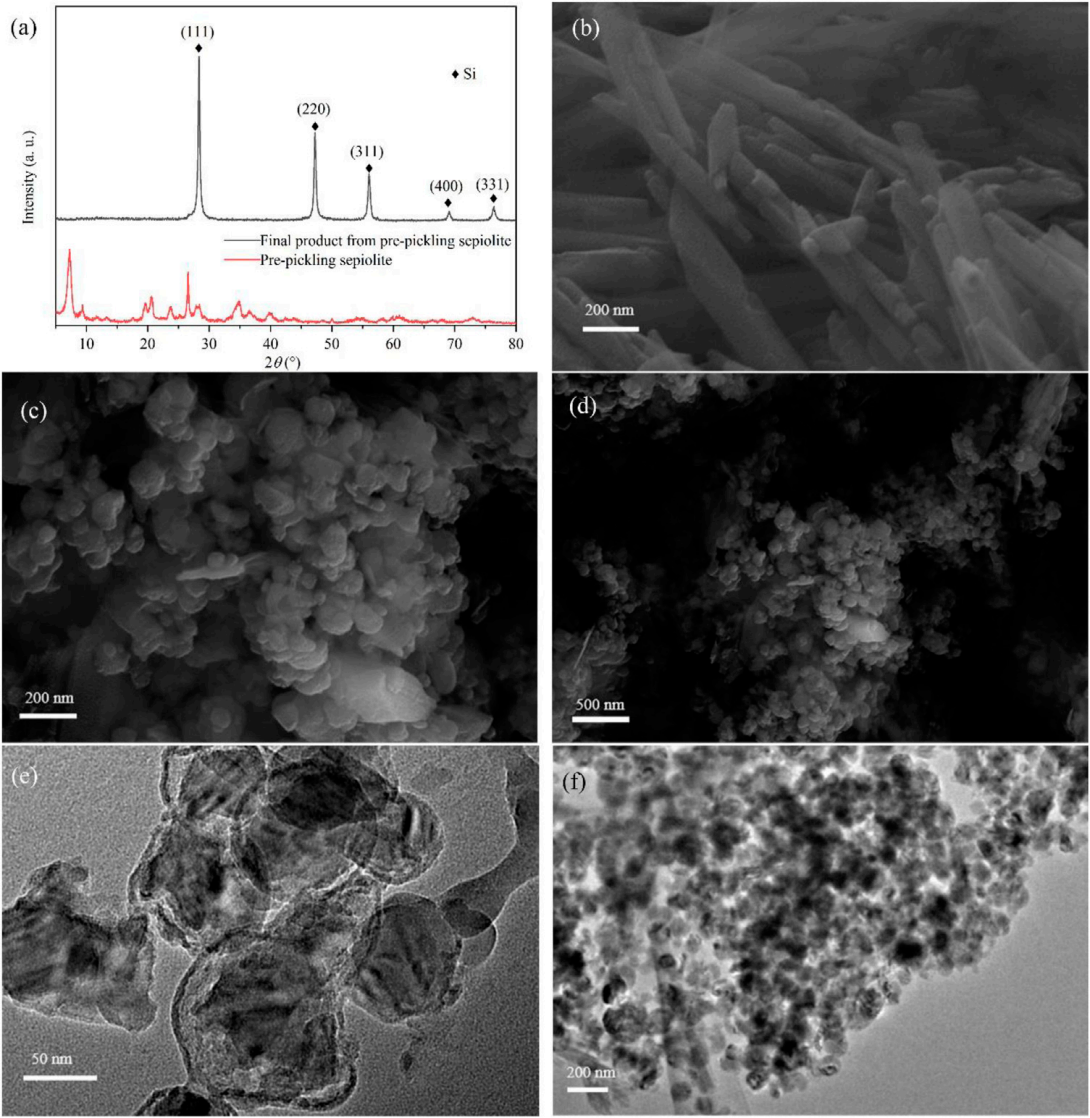
**(A)** XRD patterns of pre-pickling sepiolite and final product from pre-pickling sepiolite. **(B)** SEM of pre-pickling sepiolite showing 1D nanofiber morphology. **(C,D)** SEM and **(E,F)** TEM of the final product showing nanosphere morphology.

Some previous studies indicated that the morphology of the precursor could not be retained during low-temperature aluminothermic reduction due to its different reduction mechanism from magnesium thermal reduction (Liang et al., 2015; Zhou et al., 2016). The synergistic effect of surface energy minimization, Kirkendall effect, or Ostwald ripening could reconstruct the morphology of the final product (Liang et al., 2015; Ryu et al., 2016). In some studies, the reduced Si nanoparticles are even fused into bulk crystals, resulting in the loss of the precursor’s role as a template (Vrankovic et al., 2017; Gao et al., 2018). According to the XRD pattern of pre-pickled sepiolite (Figure 4A), there was no structure of magnesium oxide octahedron. In addition, the pre-pickling sepiolite still maintained the nanofiber structure (Figure 4B), which indicated that acid pickling would not destroy the original structure and morphology of natural sepiolite. In comparison, pre-pickling sepiolite without Mg-O octahedral structure was subjected to the same low-temperature aluminothermic reduction process at 260°C, the XRD results of the final product (Figure 4A) showed that the final product had the same Si diffraction peak as SNF, which meant SiO_2_ in pre-pickling sepiolite was completely reduced. Different from SNF, the final product showed the morphology of nanospheres according to its SEM (Figures 4C and D) and TEM (Figures 4E and F), which indicated that the nanofiber structure of natural sepiolite could not be retained during the low-temperature aluminothermic reduction process without the support of Mg-O octahedral structure. During the low-temperature aluminothermic reduction process in this study, SNF maintained a fibrous structure similar to natural sepiolite. The successful synthesis of SNF by the self-template method might be attributed to the following points: 1) The intact Mg-O octahedral layer served as the isolation band, which prevented the contact of Si nanoparticles formed in different fiber channels and inhibited the large-scale disordered binding of Si nanoparticles during the reduction process, avoided the destruction of the fibrous structure of sepiolite; 2) Adsorption of Si nanoparticles formed during reduction by a large number of adsorption sites in the fibrous structure of sepiolite weakened its disordered motion and axial displacement along the fiber pore, thus reduced the agglomeration of Si nanoparticles in the fiber channels, which was conducive to further maintaining the structure of natural sepiolite.

**FIGURE 5:**
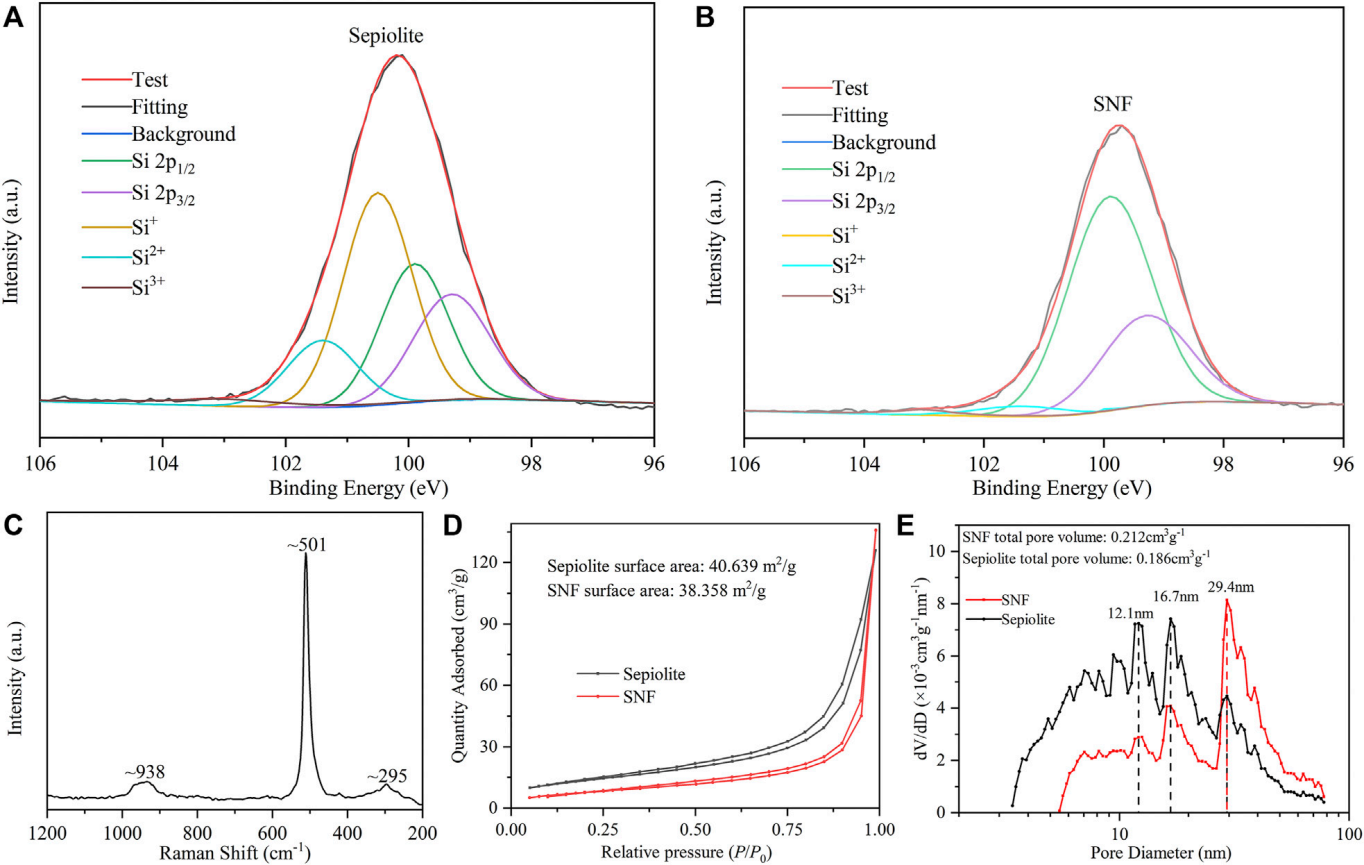
High-resolution Si 2p XPS spectrum of **(A)** natural sepiolite and **(B)** SNF. **(C)** Raman spectra of SNF. **(D)** N_2_ adsorption–desorption isotherm and **(E)** pore size distribution curve of natural sepiolite and SNF.

The distribution of the Si valence state of sepiolite before and after low-temperature aluminothermic reduction was analyzed by the Si 2p XPS spectrum. In the XPS spectrum of natural sepiolite (Figure 5A), there were two strong asymmetric peaks at ∼ 100.5 and ∼101.4 eV belonging to SiO_x_ derivatives. In addition, the asymmetric peaks composed of Si 2p_1/2_ and Si 2p_3/2_ of pure Si appeared at ∼99.9 and ∼99.3 eV. SNF showed strong asymmetric peaks at ∼99.9 and ∼99.3 eV, respectively (Figure 5B), and the intensity of the asymmetric peaks belonging to SiO_x_ derivatives decreased, which proved that both SiO_2_ and SiO_x_ derivatives were reduced to Si during the reduction process. The very weak peak at ∼ 101.4 eV was attributed to SiO_x_ derivatives, which indicated that there was an almost negligible amorphous SiO_2_ on the surface of SNF, which might be caused by the weak oxidation of Si nanocrystals in the air. Raman spectra of SNF (Figure 5C) were used to further analyze Si nanofibers obtained by low-temperature aluminothermic reduction. Compared with single crystal silicon spectra, the Raman spectra of SNF red-shifted to ∼501 cm^−1^ relative to the standard Raman spectrum of elemental silicon and widen. The same trend appeared in two broad peaks corresponding to the overtones of TA (x) and to (L) at 301 and 938 cm^−1^, which can be attributed to the decrease in Si particle size.

The natural sepiolite and SNF obtained by low-temperature aluminothermic reduction were tested by nitrogen adsorption and desorption. Isotherms of sepiolite and SNF (Figure 5D) belong to the characteristic IV type, in which the rapid increase of N_2_ adsorption capacity meant the existence of large pores. Meanwhile, the H3 hysteresis loop indicated that its pore type was a non-rigid slit-shaped pore, and the hysteresis loop was observed in the range of relative pressure *P*/*P*_*0*_ = 0.7–0.9, indicating that the mesoporous was distributed in the material, which was in good agreement with the TEM observation. Natural sepiolite had a concentrated pore size distribution at about 12.1 and 16.7 nm, respectively, indicating that it had a hierarchical pore structure. The randomness of sepiolite fiber stacking degree led to a wider pore size distribution. According to the result of pore size distribution and total pore volume in Figure 5E, SNF had similar pore size distribution as natural sepiolite, indicating that it retained the internal structure of natural sepiolite, but the concentrated pore size distribution increased from 12.1 nm to 16.7–29.4 nm. In addition, the total pore volume of SNF was larger than that of natural sepiolite, which could be attributed to the removal of mineral impurities in the sepiolite material, Mg-O octahedron, and the by-product AlOCl after low-temperature aluminothermic reduction during the pickling process to produce new pores. These pore structures provided effective accommodating space for the volume expansion during the process of deinsertion and insertion of Li^+^ in SNF anode and buffered the volume effect of the Si material, which improved the stability of the electrode structure.

**FIGURE 6:**
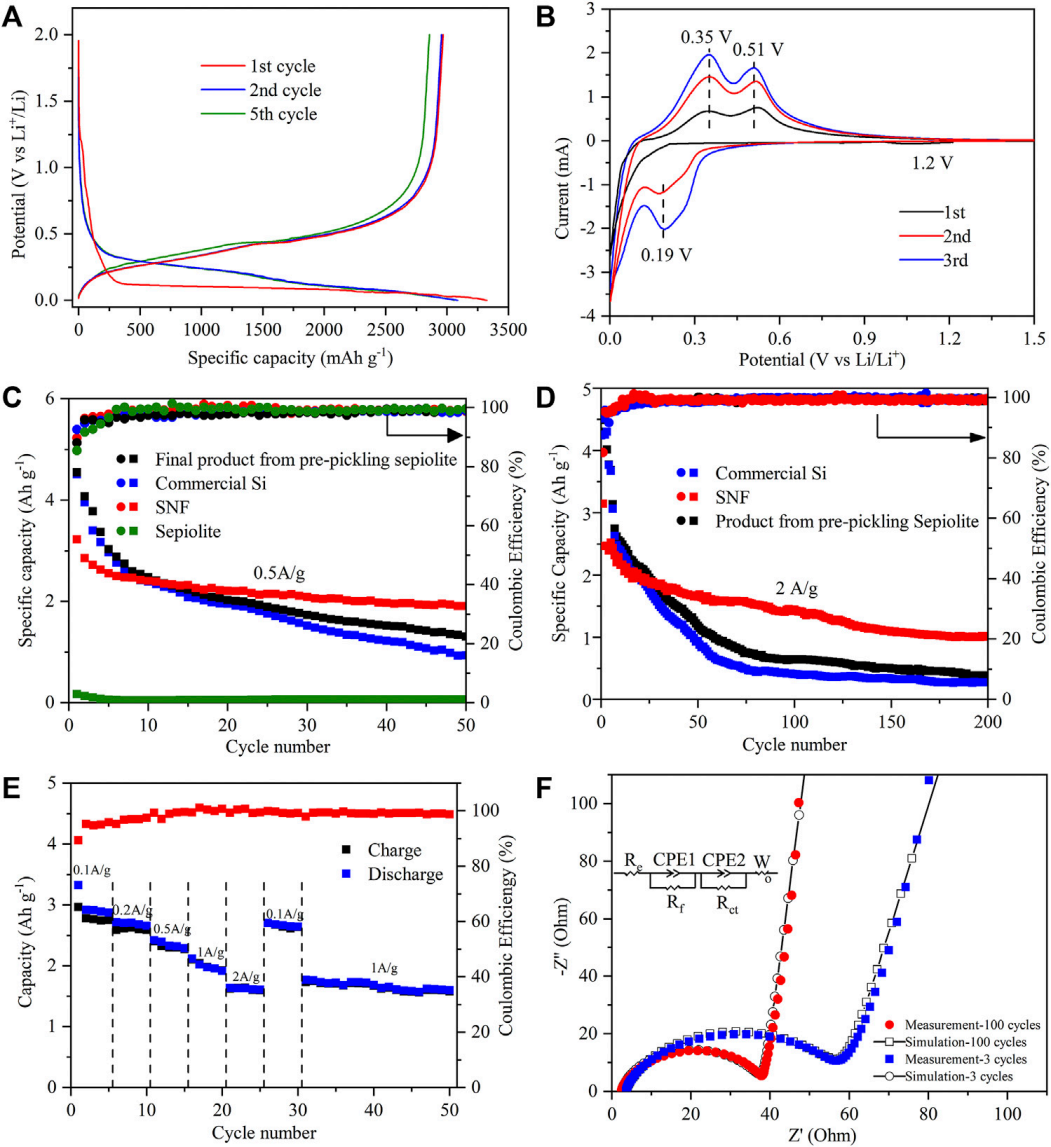
**(A)** Charge–discharge curves of SNF at a current density of 0.5 A g^−1^ between 0.001 and 2 V (vs. Li/Li^+^). **(B)** Cyclic voltammetry curves at a scan rate of 0.1 mV s^−1^ in the voltage range of 0.001–1.5 V of SNF for the first three cycles. Long-term cycling comparison of different products at **(C)** 0.5 A g^−1^ and **(D)** 2 A g^−1^. **(E)** Rate capabilities at various current densities from 0.1 to 2 A g^−1^ and **(F)** measured points with calculated lines for the impedance after 3 and 100 cycles at 2.0 A g^−1^ of SNF.

As shown in the discharge–charge curves of the SNF anode (Figure 6A), there was a voltage plateau near 1.2 V during the first lithium insertion process, but it disappeared in the subsequent cycles, which were supposed to be the formation process of SEI film. With the decrease of potential in the first lithium intercalation process, the anode entered a long voltage plateau at about 0.1 V, and the corresponding process was the first intercalation of lithium to form Li_x_Si alloy. The voltage plateau near 0.45 V on the subsequent charging curve assigned to the formation of amorphous silicon during the initial lithium deintercalation process. Starting from the second cycle, the discharge curve of SNF showed the characteristics of amorphous silicon that the lithium insertion potential platform was replaced by a slanted curve and the lithium deintercalation potential platform was almost unchanged. The first discharge/charge specific capacities of the SNF anode were 3321 mAh g^−1^ and 2968 mAh g^−1^, with the corresponding initial coulomb efficiency was 89.3%. The first cycle capacity loss might have been caused by the formation of the SEI membrane by electrolyte decomposition and the irreversible reaction between Li^+^ and active substances, which consumed a large amount of Li^+^.

The electrochemical behavior of the SNF anode was further studied by cyclic voltammetry (Figure 6B). In the first CV cycle, a wide reductive peak was observed near 1.2 V, corresponding to the formation of SEI film on the SNF surface, and this peak disappeared in the subsequent cycle, which was consistent with the results of discharge-charge curves. The sharp reductive peak below 0.13 V assigned to the Li_x_Si formation process of Li^+^ intercalation into crystal Si and two oxidative peaks in 0.35 and 0.51 V resulted from the delithiation process of the Li_x_Si phase to the amorphous Si phase. During the second CV cycle, the position of the oxidative peak was unchanged and its intensity increased. A new strong peak appeared at about 0.19 V in the reduction process, pointing to the lithiation process of amorphous Si, which confirmed the characteristics of the discharge curve. After the first two cycles, the current of peaks gradually rose with the increase of scanning times, indicating that SNF was gradually activated during the cycle.

The electrochemical properties of commercial Si, sepiolite, and the final product from pre-pickling sepiolite and SNF were tested at different current densities of 0.5 A g^−1^ (Figure 6C) and 2 A g^−1^ (Figure 6D). When the current density was 0.5 A g^−1^, the final product from pre-pickling sepiolite and commercial Si exhibited the highest initial discharge capacity (∼4500 mAh g^−1^), but the capacity began to decline rapidly after several cycles. After 50 cycles, the capacity of the final product from pre-pickling sepiolite and commercial Si remained at 1304 mAh g^−1^ and 976.4 mAh g^−1^, respectively. The reason for this phenomenon was that nano Si powder has large volume expansion and non-uniform internal stress distribution during cycles, which made the active particles easy to pulverize and lost electrical contact, leading to a rapid decline of capacity. When natural sepiolite was directly used as a negative electrode of lithium battery, the first discharge specific capacity was only 167.3 mAh g^−1^ because its main component was SiO_2_, followed by a slow decrease to a reversible capacity of 60.2 mAh g^−1^ after 50 cycles. In contrast, SNF with a one-dimensional nanofiber structure exhibited excellent cyclic stability that its first discharge specific capacity is 3150.2 mAh g^−1^ and a high capacity of 2005.4 mAh g^−1^ was still retained after 50 cycles at a high current density of 0.5 A g^−1^. When the current density increased to 2.0 A g^−1^, the capacity of SNF remained at 1017.6 mAh g^−1^ after 200 cycles (Figure 6D), while the capacity of the final product from pre-pickling sepiolite and Si was only 385.7 mAh g^−1^ and 275.4 mAh g^−1^ after 200 cycles. As a comparison, the electrochemical performance of silicon anode materials in the existing research is given in Table 2. The results show that the SNF prepared in this work has the highest capacity. The excellent performance of as-prepared SNF might result from the following factors. First, the nano size, as small as ∼100 nm, can reduce the absolute volume expansion of SNF. Meanwhile, the multi-stage pore structure in the material can effectively accommodate the volume effect of SNF during cycles. As a merit of the nanostructure, the Si fibers derived from natural sepiolite exhibited significantly high specific capacity, excluding excellent rate performance.

**TABLE 2:** Electrochemical performance of silicon anode in existing research.

Anode materials	First charge specific capacity/(mAh/g)	Initial Coulombic/% efficiency/%	Cycle umber/Capacity after cycle/(mAh g^−1^)	Current density/(A/g)
Si	2413	79.4	200/708	2
Si	3252.6	68	200/517	0.4
Si	3302.5	70.9	200/857	0.2
Si	2108	63.2	200/973	0.1
Si	2901	59.7	200/657	1
Si	3015	71.1	500/440	2

The rate performance of SNF as the anode of the lithium-ion battery was tested at rates of 0.1 A g^−1^, 0.2 A g^−1^, 0.5 A g^−1^, 1 A g^−1^, and 2 A g^−1^ (Figure 6E). As the results show, SNF had good rate performance as an anode material that with the increase of cycle rate from 0.1 A g^−1^–2A g^−1^, the discharge capacity of SNF decreased steadily from 2921.6 mAh g^−1^ in the second cycle to 1633.6 mAh g^−1^ in the 21st cycle. When the rate was back to 0.1 A g^−1^, the discharge specific capacity could restore to 2701.8 mAh g^−1^, which was 92.5% of the specific capacity of the second cycle. This phenomenon suggested that SNF had good electrochemical reversibility when used as anode material. It should be noted that when the current density suddenly switched to 2 A g^−1^, the SNF electrode provided a very stable capacity of 1633.6 mAh g^−1^, which meant that the anode material had excellent cycle stability.

In order to investigate the interface characteristics of SNF, the AC impedance of the SNF anode after different cycles was measured (Figure 6F). It can be seen from the figure that the diameter of the high-frequency semicircle of the electrode after 100 cycles was smaller than that after 3 cycles, which indicated that the electrode had lower SEI film impedance and charge transfer impedance. In addition, the slope of the low-frequency line increased after the cycle, which represented that the diffusion resistance of lithium-ion in the electrode material decreased gradually with the lithium-ion intercalation/deintercalation cycle. The fitting data based on the equivalent circuit shown in the figure also confirmed the above results. R_e_, R_f_, and R_ct_ corresponded to the electrolyte resistance, SEI film impedance, and charge transfer impedance, respectively (Chen et al., 2018; Xiao et al., 2020). The resistance values relating to different cycle numbers are listed in Table 3.

**TABLE 3:** Fitting parameters of electrochemical impedance spectroscopy for SNF after 3 and 100 cycles.

Cycle number	R_e_ [Ω]	R_f_ [Ω]	R_ct_ [Ω]
3	3.4	2.7	54.5
100	2.7	2.5	38.2

## Conclusion

In summary, SNF was successfully synthesized from natural sepiolite by a low-cost and environmentally friendly synthesis method. Without complex pre/post-treatment, the 1D structure of SNF was maintained during low-temperature aluminothermic reduction. In addition, sepiolite without Mg-O octahedral structure was subjected to the same low-temperature aluminothermic reduction process. By comparing the morphology of SNF and final product from pre-pickling sepiolite, the significant role of Mg-O octahedron in maintaining the fibrous structure of sepiolite during the low-temperature aluminothermic reduction process was verified by experiments for the first time. When SNF was used as an anode for lithium-ion batteries, it exhibited excellent electrochemical performance, including cycling stability (2005.4 mAh g^−1^ at 0.5 A g^−1^ after 50 cycles and 1197.6 mAh g^−1^ at 2 A g^−1^ after 200 cycles) and remarkable rate capability (the discharge capacity of SNF decreased steadily from 2921.6 mAh g^−1^ at 0.5 A g^−1^ to 1633.6 mAh g^−1^ at 2 A g^−1^). Our work provided a new strategy for the synthesis of commercial high-capacity nanostructured Si anode in LIBs from natural clay minerals.

## Data Availability

The original contributions presented in the study are included in the article/Supplementary Material; further inquiries can be directed to the corresponding author.
